# Supplementary material for: Optical characterization of red blood cells from individuals with sickle cell trait and disease in Tanzania using quantitative phase imaging
Source: Sci Rep. 2016 Aug 22;6:31698. doi: 10.1038/srep31698 (PMC4992839; doi:10.1038/srep31698)
Supplement: Supplementary Information [file srep31698-s1.doc]

Supplementary information

Optical characterization of red blood cells from individuals with sickle cell trait and disease in Tanzania using quantitative phase imaging

**JaeHwang Jung^1,*^, Lucas E. Matemba^2,*^, KyeoReh Lee^1^, Paul E. Kazyoba^3^, Jonghee Yoon^1^,
Julius J. Ma ssaga^3^, Kyoohyun Kim^1^, Dong-Jin Kim^4,+^, and YongKeun Park^1,5,+^**

^1^ Department of Physics, Korea Advanced Institute of Science and Technology, Daejeon 34141, Republic of Korea

^2^ National Institute for Medical Research, P.O. Box 476, Morogoro, Tanzania

^3^ National Institute for Medical Research, 3 Barack Obama Drive, P.O. Box 9653, 11101 Dar es Salaam, Tanzania

^4^ Nelson Mandela African Institution of Science and Technology, School of Life Science and Bioengineering, P.O. Box 447 Arusha, Tanzania

^5^TomoCube, Inc., Daejeon 34051, Republic of Korea

*These authors contributed equally to this work.

Correspondence and requests for materials should be addressed to Y.K.P (email: [yk.park@kaist.ac.kr](mailto:yk.park@kaist.ac.kr)) and D.J.K. (email: [djkim.nmaist@gmail.com](mailto:djkim.nmaist@gmail.com)).

1

Supplementary Table S1. Measured RBC properties for each donor. Average aspect ratios, membrane fluctuations, Hb contents and dimple curvatures of the individuals with healthy, SCT and SCD. The values are presented as mean ± standard deviation. The values in “Avg.” rows indicate means and standard deviation of mean values for each group.

| Type | Donor  (Number of cells) | Aspect Ratio | | Fluctuation (nm) | | Hb contents (pg) | | Dimple Curvature (µm^-1^) | |
| --- | --- | --- | --- | --- | --- | --- | --- | --- | --- |
| Healthy | 1 (31) | 0.890± 0.115 | | 46.93 ± 6.72 | | 28.52 ± 4.48 | | -0.169 ± 0.138 | |
|  | 2 (24) | 0.841 ± 0.101 | | 51.73 ± 10.38 | | 30.28 ± 3.28 | | -0.052 ± 0.240 | |
|  | 3 (35) | 0.911 ± 0.068 | | 41.99 ± 5.99 | | 35.08 ± 3.86 | | -0.021 ± 0.118 | |
|  | 4 (22) | 0.847 ± 0.109 | | 40.25 ± 5.17 | | 27.00 ± 4.82 | | -0.094 ± 0.091 | |
|  | 5 (37) | 0.897 ± 0.104 | | 47.22 ± 7.08 | | 31.74 ± 3.71 | | -0.253 ± 0.124 | |
|  | Avg. | 0.878 ± 0.031 | | 45.63 ± 4.57 | | 30.53 ± 3.11 | | -0.118 ± 0.094 | |
| SCT | 1 (28) | 0.897 ± 0.080 | | 40.78 ± 5.01 | | 32.79 ± 4.21 | | -0.133 ± 0.103 | |
|  | 2 (20) | 0.910 ± 0.047 | | 36.89 ± 3.93 | | 28.72 ± 3.05 | | -0.066 ± 0.097 | |
|  | 3 (35) | 0.927 ± 0.037 | | 41.88 ± 4.53 | | 26.55 ± 3.06 | | -0.195 ± 0.110 | |
|  | 4 (31) | 0.857 ± 0.083 | | 37.41 ± 6.62 | | 23.12 ± 2.93 | | -0.205 ± 0.084 | |
|  | 5 (32) | 0.866 ± 0.083 | | 41.01 ± 5.42 | | 27.85 ± 2.19 | | -0.208 ± 0.130 | |
|  | 6 (31) | 0.911 ± 0.052 | | 38.62 ± 5.08 | | 27.45 ± 3.08 | | -0.172 ± 0.112 | |
|  | 7 (47) | 0.902 ± 0.066 | | 41.78 ± 5.00 | | 28.52 ± 4.29 | | -0.160 ± 0.112 | |
|  | 8 (30) | 0.883 ± 0.073 | | 38.62 ± 6.15 | | 28.67 ± 3.52 | | -0.157 ± 0.016 | |
|  | 9 (32) | 0.903 ± 0.057 | | 42.80 ± 5.61 | | 30.50 ± 3.45 | | -0.179 ± 0.117 | |
|  | Avg. | 0.895 ± 0.023 | | 39.98 ± 2.13 | | 28.24 ± 2.65 | | -0.164 ± 0.044 | |
| SCD | (Number of ISC/RSC) | ISC | RCS | ISC | RCS | ISC | RCS | ISC | RCS |
|  | 1 (5/18) | 0.422  ± 0.122 | 0.705  ± 0.172 | 26.77  ± 1.61 | 44.24  ± 7.52 | 28.50  ± 5.96 | 35.62  ± 4.28 | 0.005  ± 0.220 | -0.198  ± 0.273 |
|  | 2 (4/15) | 0.467  ± 0.108 | 0.831  ± 0.137 | 26.06  ± 3.39 | 41.15  ± 5.94 | 24.99  ± 5.17 | 29.39  ± 2.83 | -0.155  ± 0.096 | -0.263  ± 0.131 |
|  | 3 (1/16) | 0.466  ± 0.000 | 0.888  ± 0.087 | 27.11  ±0.00 | 40.18  ± 6.64 | 28.32  ± 0.00 | 27.35  ± 5.50 | -0.209  ± 0.000 | -0.243  ± 0.100 |
|  | 4 (4/18) | 0.441  ± 0.022 | 0.807  ± 0.168 | 27.37  ± 1.13 | 41.35  ± 7.28 | 23.68  ± 2.27 | 31.73  ± 4.18 | -0.050  ± 0.126 | -0.168  ± 0.119 |
|  | 5 (2/21) | 0.532  ± 0.033 | 0.820  ± 0.122 | 30.43  ± 2.18 | 40.23  ± 5.38 | 27.30  ± 5.36 | 29.55  ± 4.50 | -0.155  ± 0.091 | -0.228  ± 0.094 |
|  | 6 (6/19) | 0.473  ± 0.050 | 0.783  ± 0.119 | 25.98  ± 3.97 | 38.99  ± 6.94 | 33.66  ± 1.85 | 34.85  ± 4.03 | -0.085  ± 0.188 | -0.143  ± 0.147 |
|  | 7 (4/25) | 0.470  ± 0.065 | 0.861  ± 0.069 | 18.11  ± 3.43 | 39.05  ± 3.53 | 26.38  ± 5.08 | 30.93  ± 5.24 | -0.021  ± 0.147 | -0.137  ± 0.096 |
|  | 8 (8/22) | 0.380  ± 0.085 | 0.714  ± 0.141 | 31.18  ±2.71 | 39.67  ±6.53 | 30.75  ± 4.82 | 28.29  ± 3.67 | 0.067  ± 0.289 | -0.221  ± 0.148 |
|  | Avg. | 0.456  ± 0.044 | 0.801  ± 0.065 | 26.67  ± 4.00 | 40.57  ± 1.74 | 27.95  ± 3.18 | 30.96  ± 2.97 | -0.076  ± 0.093 | -0.200  ± 0.047 |

Supplementary Figure S1. Aspect ratios of RBCs.

Individual cell data and mean values for individual donors are presented. Statistical tests are performed considering each mean value of individual donors as a single data. Mann–Whitney U-tests are applied on SCT, ISC, and RSC groups against the Healthy group because the distribution of aspect ratios would not follow normal distributions (**: *p*<0.01; ***: *p*<0.001). The results suggest the average shapes of the SCD RBCs, both ISCs and RSCs, are different from the average shapes of the healthy RBCs while the SCT RBCs have indistinguishable shapes with healthy RBCs.

Circles present individual cell values. Squares and short horizontal bars present means and standard deviations of measured values for each donor, respectively. Long bars and shaded areas present means and standard deviations of mean values for each groups, respectively, which are also presented in numerical values. Black, red, blue and green colors indicate Healthy, SCT, ISC, and RSC groups.

Supplementary Figure S2. Membrane curvatures of RBCs.

Individual cell data and mean values for individual donors are presented. Student’s *t*-tests are performed considering each mean value of individual donors as a single data (**: *p*<0.01). Statistical significance implies the mean curvatures of SCT and SCD RBCs may not be different from the mean curvature of healthy RBCs. Within SCD RBCs, however, ISCs have significantly flat curvatures compared to the membrane curvatures of RSCs. This may imply that the ISCs partially lose dimple curvatures during a sickling process.

Circles present individual cell values. Squares and short horizontal bars present means and standard deviations of measured values for each donor, respectively. Long bars and shaded areas present means and standard deviations of mean values for each groups, respectively, which are also presented in numerical values. Black, red, blue and green colors indicate Healthy, SCT, ISC, and RSC groups.

Supplementary Figure S3. Hb contents of RBCs.

Individual cell data and mean values for individual donors are presented. Student’s *t*-tests are performed considering each mean value of individual donors as a single data. However, no significant difference is observed between groups.

Circles present individual cell values. Squares and short horizontal bars present means and standard deviations of measured values for each donor, respectively. Long bars and shaded areas present means and standard deviations of mean values for each groups, respectively, which are also presented in numerical values. Black, red, blue and green colors indicate Healthy, SCT, ISC, and RSC groups.

Supplementary Figure S4. Membrane fluctuation levels of RBCs.

Individual cell data and mean values for individual donors are presented. Student’s *t*-tests are performed considering each mean value of individual donors as a single data (*: *p*<0.05; **: *p*<0.01; ***: *p*<0.001). SCT, ISC, and RSC groups shows statistical significances.

Circles present individual cell values. Squares and short horizontal bars present means and standard deviations of measured values for each donor, respectively. Long bars and shaded areas present means and standard deviations of mean values for each groups, respectively, which are also presented in numerical values. Black, red, blue and green colors indicate Healthy, SCT, ISC, and RSC groups.

Supplementary Figure S5. Elliptical fitting of cell boundary. (left) ISCs (right) RSCs

The long and short axes of a cell were found by fitting an ellipse to the cell boundary, as shown in Figure S5. The fitting was performed by a custom-base script in MatLab.
